# Indexing of Iranian Publications in Well-known Endodontic Textbooks: A Scientometric Analysis

**DOI:** 10.7508/iej.2016.03.002

**Published:** 2016-05-01

**Authors:** Sina Kakooei, Mahshid Mostafavi, Masoud Parirokh, Saeed Asgary

**Affiliations:** a *Endodontology Research Center, Kerman University of Medical Sciences, Kerman, Iran; *; b * Leishmaniasis Research Center, Kerman University of Medical Sciences, Kerman, Iran; *; c * Oral and Dental Diseases Research Center, Kerman University of Medical Sciences, Kerman, Iran; *; d * Iranian Center for Endodontic Research, Research Institute of Dental Sciences, Dental School, Shahid Beheshti University of Medical Sciences, Tehran, Iran*

**Keywords:** Dental Pulp, Endodontics, Index, Ingle, Iranian Publications, Pathways of the Pulp, Quote, Scientometric, Textbooks

## Abstract

**Introduction:**

Quoting an article in well-known textbooks is held as a credit for that paper. The numbers of Iranian publications mentioned in endodontic textbooks have increased during recent years. The aim of this investigation was to evaluate the number of Iranian articles quoted in eminent endodontic textbooks.

**Methods and Materials:**

Three known textbooks (*Ingle’s Endodontics, Seltzer and Bender’s Dental Pulp* and *Cohen’s Pathways of the Pulp*) were chosen and all the editions of the textbooks since 2000 were investigated for quoted Iranian publications. Only Iranian authors with affiliations from a domestic university were chosen. All references at the end of each chapter were read by hand searching, and results were noted. The trend and percentage of Iranian publications in different editions of the textbooks were also calculated. The number of citations of these publications in Google Scholar and Scopus databases were also obtained.

**Results:**

The number of Iranian publications in all well-known textbooks have notably increased since 2000. The number and percentage of Iranian publications in the latest edition of Cohen’s Pathways of the Pulp was higher compared to other textbooks as well as the previous edition of the same text.

**Conclusion:**

Number and percentage of Iranian publications in the field of endodontics in all three textbooks have remarkably increased since 2000.

## Introduction

One of the most important aims of publishing scientific articles is to provide a reliable source of information for readers. Although performing laboratory and animal studies is necessary to provide basic information regarding the efficacy and safety of dental materials and techniques, nowadays the most leading dental journal are emphasizing on evidence-based investigations in order to provide reliable results for clinical practice [1, 2]. 

Textbooks are important documents in scientific field. Text book contributors are usually among well-known high reputation scientists and for that reason the chapters that have been prepared by them would be a reliable source for the readers. Therefore, quoting an article in a text book is assumed to be held as a credit for the authors. It has been reported that the science production in dentistry has faced a 5.68% growth from 2000 to 2009 [3]. Because of the encouraging policy of national universities, Iranian researchers prefer to publish their papers in international English journals. Therefore, there is a tendency to increase international publications of the Iranian endodontists. 

Endodontics is one of the well-organized fields of dentistry in Iran. *Iranian Association of Endodontics* (*IAE*) is a nongovernmental association that has been founded since 1995. 

**Figure 1:**
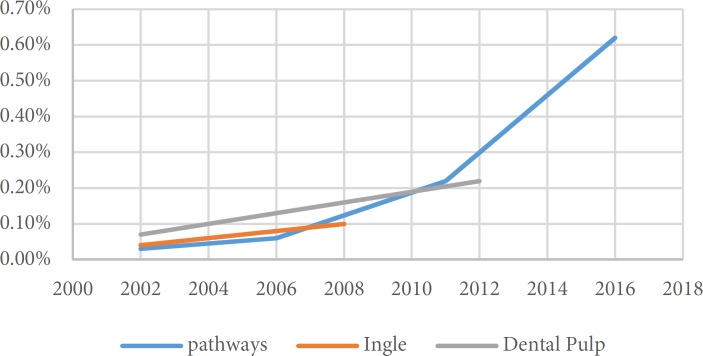
Trend of Iranian publication in different text book since 2000

Apart from holding annual congresses since 1997, *IAE* is the holder of *Iranian Endodontic Journal* *(IEJ)* which has been publishing endodontic papers since 2006. *IEJ* is indexed in a number of data bases including Scopus and PubMed. 

Scientific authority is an important issue for both Iranian universities and *IAE*. The aim of the present study is to evaluate the trend of appearance of the publications from Iranian authors in well-known endodontic textbooks.

## Materials and Methods

Three textbooks which are references for Iranian board certificate (including *Ingle’s Endodontics*, *Seltzer and Bender’s Dental Pulp* and *Cohen’s Pathways of the Pulp*) were chosen to be evaluated (Table 1). In addition, in order to evaluate the trend of Iranian endodontists quotes in these books, all editions of the books since 2000 had been evaluated. The reference part of all chapters of these books had been evaluated through hand searching by two independent reviewers. All Iranian names had been collected and then reviewed by the third reviewer. Only the articles that their first author was Iranian with domestic affiliation were chosen. By using Google Scholar, PubMed and Scopus data bases, the affiliation and the number of each cited articles were extracted. If a paper was cited several times in the same text book, only one quote was taken into account. University of the authors was also considered. The percentage of Iranian endodontists quotes in each text book was calculated by dividing the number of the Iranian papers by the total number of references that appeared in those books.

## Results

**Figure 2:**
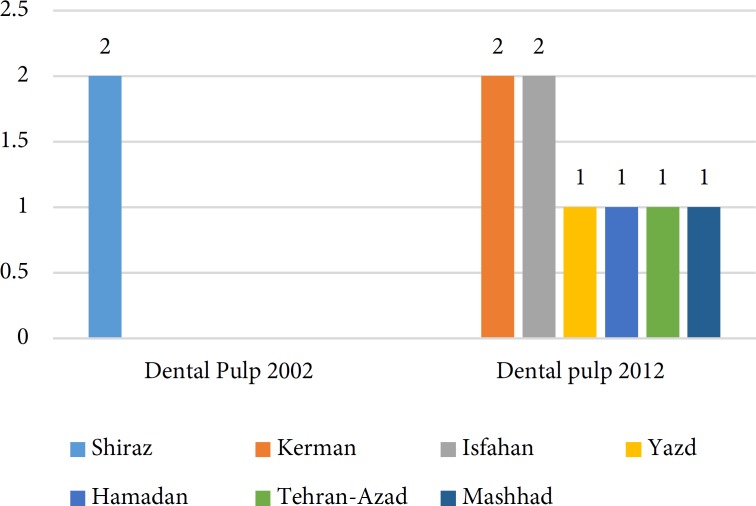
Universities that their publications had been cited in Seltzer and Bender’s Dental Pulp

**Figure 3:**
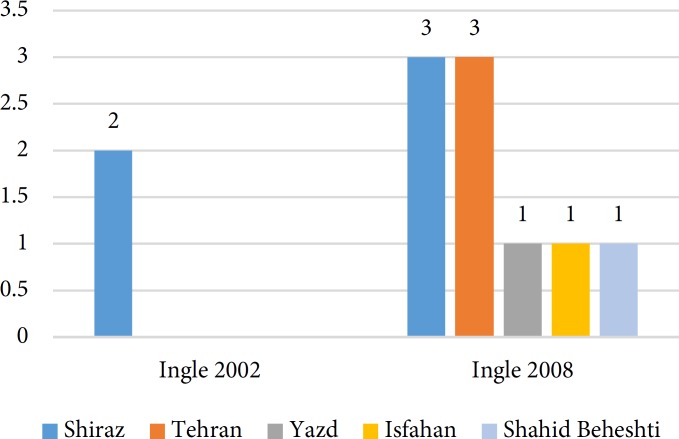
Universities that their publications had been cited in Ingle’s Endodontics

**Figure 4:**
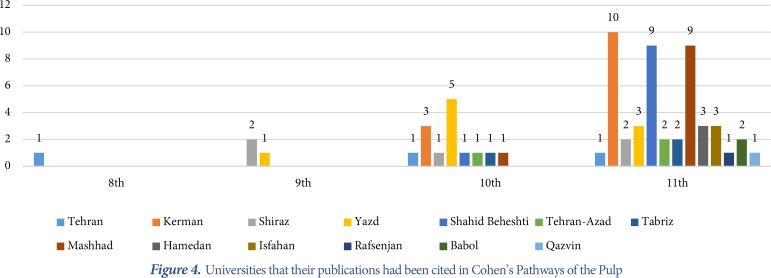
Universities that their publications had been cited in Cohen’s Pathways of the Pulp

A total of 64 papers have been found in separate versions of the textbooks [4-66]. Since 2000, *Cohen’s Pathways of the Pulp* was reprinted 4 times, whereas *Ingle’s endodontics* and *Bender and Seltzer’s Dental Pulp* had been reprinted twice within the same time period. Table 2 shows the number of the Iranian papers cited in consecutive publications in different editions of the textbooks. Figure 1 shows the trend of Iranian authors’ publications that were cited in different textbooks. The type of quoted papers in descending order was randomized-controlled clinical trials, *in vitro* studies and review papers (Table 3). Several investigations have been quoted in different editions of the textbooks (Table 4). The trend of publications in *Cohen’s Pathways of the Pulp* showed that the number of citations has increased from 0.003 in 2002 to 0.62 in 2016. Figures 2 to 4 show that articles published by endodontic departments of some universities’ had been more quoted.

## Discussion

The results of the present study showed that the citation of the articles that had been written by Iranian authors in well-known textbooks have notably increased during recent years. In the present study, three well known textbooks (Table 1) were used to evaluate the scientific authority of Iranian endodontists because these books were chosen as the reference books of postgraduate programs in Iran [67]. In this study, only the papers with their first author being affiliated from one of the domestic universities were chosen, because previous investigations in sicentometric field also considered this criterion [68]. 

**Table 1:** List of the investigated textbooks

**Book name **	**Edition **	**Editors**	**Year **	**Publisher**
**Pathways of the Pulp**	Eighth	Stephen Cohen Richard C. Burns	2002	Mosby Elsevier
**Pathways of the Pulp**	Ninth	Stephen Cohen Kenneth M. Hargreaves	2006	Mosby Elsevier
**Cohen's Pathways of the Pulp**	Tenth	Kenneth M. Hargreaves Stephen S. Cohen, Louis H. Berman	2011	Mosby Elsevier
**Cohen's Pathways of the Pulp**	Eleventh	Kenneth M. Hargreaves Louis H. Berman	2016	Mosby Elsevier
**Ingle’s Endodontics**	Fifth	John I. Ingle Leif K. Bakland	2002	B.C. Decker
**Ingle’s Endodontics**	Sixth	John I. Ingle Leif K. Bakland Craig Baumgartner	2008	B.C. Decker
**Seltzer and Bender’s Dental Pulp**	First	Kenneth M. Hargreaves Harold E. Goodis	2002	Quintessence Pub Co
**Seltzer and Bender’s Dental Pulp**	Second	Kenneth M. Hargreaves Harold E. Goodis	2012	Quintessence Pub Co

The trend of Iranian authors’ publications that were quoted in these textbooks was particularly promoted in the recent 11^th^ edition of *Cohen’s Pathways of the Pulp*. *Cohen’s Pathways of the Pulp* was also the most frequently-revised text book compared to the two other textbooks (Tables 2 and 3). 

Most Iranian authors’ papers that were quoted in the latest edition of *Cohen’s Pathways of the Pulp* were randomized clinical trials followed by *in vitro* and review papers (Table 4). Nowadays, high level of evidence investigations are being encouraged to be performed because of their high reliability for clinical practice [1, 2]. 

**Table 2:** Number of Iranian publications that had been cited in different textbooks

**Book name **	**Edition **	**Year **	**All references (N)**	**Iranian citations (%)**
**Pathways of the Pulp**	Eighth	2002	3063	1 (0.03)
**Pathways of the Pulp**	Ninth	2006	4830	3 (0.06)
**Cohen's Pathways of the Pulp**	Tenth	2011	6168	14 (0.22)
**Cohen's Pathways of the Pulp**	Eleventh	2016	7641	48 (0.62)
**Ingle’s Endodontics**	Fifth	2002	4164	2 (0.04)
**Ingle’s Endodontics**	Sixth	2008	8926	9 (0.1)
**Seltzer and Bender’s Dental Pulp**	First	2002	2756	2 (0.07)
**Seltzer and Bender’s Dental Pulp**	Second	2012	3499	8 (0.22)

**Table 3:** Type of Iranian articles that had been cited in different textbooks

**Book (year)**	**Systematic review**	**Randomized clinical trial**	**Clinical study**	**Review**	**Case series**	**Case report**	***In vitro***	**Animal studies**	**Total**
**Dental Pulp (2002)**	0	0	0	1	0	0	1	0	2
**Dental Pulp (2012)**	1	4	1	2	0	0	0	0	8
**Ingle (2002)**	0	0	0	0	0	0	2	0	2
**Ingle (2008)**	0	4	0	1	0	0	4	0	9
**Pathways (2002)**	0	1	0	0	0	0	0	0	1
**Pathways (2006)**	0	0	0	0	0	0	3	0	3
**Pathways (2011)**	1	2	0	4	0	1	4	2	14
**Pathways (2016)**	1	17	1	10	2	2	12	3	48
**Total**	3	28	2	18	2	3	26	5	87

**Table 4:** Number of Iranian publications’ citation in Scopus and Google Scholar

**First author**	**Pathways**				**Ingle**				**Dental Pulp**				**Google Scholar**		**Scopus**
	**2002**	**2006**	**2011**	**2016**	**2002**		**2008**		**2002**		**2012**				
**Abbasipour F [** 4 **]**				×										7	3
**Amini F [** 5 **]**												×		17	9
**Asgary S ** **[** 6 **]**								×						270	112
**Asgary S ** **[** 7 **]**				×										46	23
**Asgary S ** **[** 8 **]**				×										41	21
**Asgary S** **[** 9 **]**				×										41	12
**Asgary S ** **[** 10 **]**				×										54	NA
**Asgary S ** **[** 11 **]**				×										151	81
**Ashraf H ** **[** 12 **]**				×										27	18
**Azar NG ** **[** 13 **]**		×	×											109	33
**Birang R [** 14 **]**												×		10	7
**DaneshkazemiA R ** **[** 15 **]**		×	×											33	13
**Dastmalchi N ** **[** 16 **]**				×										20	9
**Eghbal MJ ** **[** 17 **]**				×										83	44
**Eskandarizadeh A ** **[** 18 **]**				×										44	21
**Farhad A[** 19 **]**												×		98	36
**Ghoddusi J ** **[** 20 **]**				×										47	16
**Homayouni H** ** [** 21 **]**				×										3	0
**Jafarzadeh H [** 22 **]**												×		106	29
**Jafarzadeh H** ** [** 23 **]**				×										42	13
**Jafarzadeh H** ** [** 24 **]**				×										27	16
**Jafarzadeh H** ** [** 25 **]**			×	×										68	28
**Jalalzadeh SM [** 26 **]**				×										24	15
**Javaheri HH ** **[** 27 **]**			×	×										99	37
**Kaviani N ** **[** 28 **]**				×										11	2
**Khademi A ** **[** 29 **]**				×										156	76
**Khademi AA** ** [** 30 **]**				×										14	5
**Khayat A ** **[** 31 **]**						×		×		×				323	146
**Khedmat S** **[** 32 **]**				×										41	24
**Kuzekanani M** ** [** 33 **]**				×										5	NA
**Mehrvarzfar P** ** [** 34 **]**			×	×										21	9
**Memarpour M** ** [** 35 **]**				×										2	2
**Modaresi J[** 36 **]**			×	×										61	30
**Modaresi J** **[** 37 **]**			×	×										29	13
**Mohammadi Z** ** [** 38 **]**				×										261	125
**Mohammadi Z** ** [** 39 **]**				×										162	78
**Mohammadi Z ** **[** 40 **]**			×	×										146	54
**Mohammadi Z ** **[** 41 **]**			×											9	NA
**Moosavi H ** **[** 42 **]**				×										15	3
**Mortazavi M** ** [** 43 **]**								×						103	44
**Mozayeni MA** ** [** 44 **]**				×										62	26
**Namazi MR** ** [** 45 **]**			×	×										11	6
**Nekoofar MH** ** [** 46 **]**								×						39	17
**Nekoofar MH** ** [** 47 **]**								×						152	85
**Nematollahi H** ** [** 48 **]**				×										3	1
**Nosrat A ** **[** 49 **]**				×										50	23
**Parirokh M ** **[** 50 **]**				×										44	29
**Parirokh M ** **[** 51 **]**				×										7	3
**Parirokh M ** **[** 52 **]**				×										7	5
**Parirokh M ** **[** 53 **]**				×										33	13
**Parirokh M ** **[** 54 **]**				×										509	273
**Parirokh M ** **[** 55 **]**				×										534	279
**Parirokh M ** **[** 56 **]**			×	×										7	2
**Parirokh M ** **[** 57 **]**			×											95	55
**Partovi M ** **[** 58 **]**				×										20	13
**Ravanshad S** ** [** 59 **]**						×		×						52	22
**Ravanshad S** ** [** 60 **]**		×												31	NA
**Sadeghein A[** 61 **]**		×						×						17	6
**Shahi S ** **[** 62 **]**								×						12	6
**Shahi S** **[** 63 **]**						×		×						11	3
**Shahrami FZ** ** [** 64 **]**								×						4	2
**Shahravan A [** 65 **]**				×		×						×		175	81
**Tabarsi B ** **[** 66 **]**						×								81	48
**Zarei M ** **[** 67 **]**						×								10	7

The percentage of the Iranian authors’ papers that were quoted in *Ingle’s endodontics* were lower compared to the two other textbooks. The reason of this difference may be the year of publication of these books because the latest publication of *Ingle’s endodontics* was in 2008, whereas the latest publication year of the *Cohen’s Pathways of the Pulp* and *Bender and Seltzer’s Dental Pulp* is 2016 and 2012, respectively. The number of published papers from Iranian authors in the field of endodontics has increased in recent years [69, 70]. 

## Conclusion

The current study shows positive trend of Iranian endodontic articles quoted in three endodontic textbooks.
